# A proteomics study to explore the role of adsorbed serum proteins for PC12 cell adhesion and growth on chitosan and collagen/chitosan surfaces

**DOI:** 10.1093/rb/rby017

**Published:** 2018-07-17

**Authors:** Xiaoying Lü, Heng Zhang, Yan Huang, Yiwen Zhang

**Affiliations:** 1 State Key Laboratory of Bioelectronics, School of Biological Science and Medical Engineering, Southeast University, Nanjing, P.R. China; 2 SQ Medical Device Co., Ltd., Nanjing, P.R. China

**Keywords:** chitosan and collagen/chitosan film, cell adhesion and growth, protein adsorption, proteomics/bioinformatics

## Abstract

The aim of this article is to apply proteomics in the comparison of the molecular mechanisms of PC12 cell adhesion and growth mediated by the adsorbed serum proteins on the surfaces of chitosan and collagen/chitosan films. First, the chitosan and the collagen/chitosan films were prepared by spin coating; and their surface morphologies were characterized by scanning electron microscopy, X-ray energy dispersive spectroscopy, contact angle measurement and Fourier transform infrared spectroscopy. Subsequently, cell proliferation experiments on two materials were performed and the dynamic curves of protein adsorption on their surfaces were measured. Then, proteomics and bioinformatics were used to analyze and compare the adsorbed serum proteins on the surfaces of two biomaterials; and their effects on cell adhesion were discussed. The results showed that the optimum concentration of chitosan film was 2% w/v. When compared with chitosan film, collagen/chitosan film promoted the growth and proliferation of PC12 cells more significantly. Although the dynamic curves showed no significant difference in the total amount of the adsorbed proteins on both surfaces, proteomics and bioinformatics analyses revealed a difference in protein types: the chitosan surface adsorbed more vitronectin whereas collagen/chitosan surface adsorbed more fibronectin 1 and contained more cell surface receptor binding sites and more Leu-Asp-Val sequences in its surface structure; the collagen/chitosan surface were more conducive to promoting cell adhesion and growth.

## Introduction

Natural polymers play an important role in biomaterials due to their good biocompatibility and biodegradability. Chitosan and collagen are two of the most widely-used natural polymeric materials in the biomaterials. Natural alkaline polysaccharide chitosan (chitosan) is an amino polysaccharide, and is the second largest biological resource on earth after plant fiber. Due to its excellent biocompatibility, biodegradability, physiological activity and film-forming property, chitosan has become a widespread biomaterial in the biomedical field [1]. However, some studies showed that a pure chitosan film have a certain inhibitory effect on cell adhesion and proliferation. The reason is that the presence of primary amine of glucosamine residue makes chitosan as a pH-responsive polycation; a small change in environmental pH can affect protein adsorption [2], thereby affecting cell adhesion. Currently, a large number of cell biocompatibility studies on chitosan have been reported and are mainly performed on film, including pure chitosan film and chitosan blends such as collagen/chitosan film [3, 4].

Collagens are triple helical proteins composed of alpha chains [5]. As one of the most basic components of extracellular matrix (ECM), collagen forms the skeletal structure of the ECM supports cell’s anchoring, and provides a favorable microenvironment for cell growth and proliferation [6]. Due to its good biocompatibility, low antigenicity and good biodegradability, collagen has a wide range of applications as a hemostatic material, a wound repair material, a skin substitute, a bone regeneration scaffold, a nerve tissue repair, a drug carrier material and so on [1]. Chitosan and collagen have good miscibility [7]. And it has been proved that the chitosan-collagen blends provided better biocompatibility than a pure chitosan film [8, 9].

Current studies confirmed that the biocompatibility of collagen/chitosan film was better than that of chitosan at the cellular level. However, no literature has reported the explanation ‘why’ the collagen/chitosan film has better cellular compatibility. Upon implantation of biomaterials in the body, cell adhesion and growth is preceded by protein adsorption and mediated by not a single type of adsorbed proteins but rather multiple types. Thus the types, amounts, and conformations of adsorbed proteins directly determine the subsequent cellular response. Therefore, to deeply investigate the adsorption behavior of proteins on different biomaterial surfaces is of significant importance in studying cell adhesion, growth and proliferation on material surfaces. Commonly-used methods, such as isotope labeling, iodine labeling, ultraviolet (UV)/visible spectrophotometry, infrared (IR) spectroscopy and biosensor approach [10–12], previously focused on the adsorption behavior of a single or a few specific proteins. However, biomaterials in actual use come into contact with a complex protein environment (e.g. blood) with a wide variety of proteins, whose adsorption behaviors are very complicated. The development of proteomics and bioinformatics has made it possible to study the complex protein adsorption layers high throughput.

Proteomics utilizes protein separation techniques such as 2D gel electrophoresis, 2D fluorescence difference gel electrophoresis, high-performance liquid chromatography and combines protein identification techniques such as matrix-assisted laser desorption/ionization imaging mass spectrometry (MS) and electrospray ionization MS to identify all the proteins expressed in the complex protein layers, cells or tissues. Bioinformatics provides powerful tools to solve the challenges of analyzing the large amount of data generated by proteomics, thus enabling qualitatively and quantitatively analysis of various proteins within complex compositions and their influence on cell behaviors during adhesion. At present, proteomics is mainly used to identify the differentially expressed proteins in cells or tissues under the effect of external factors. Although a few literatures had reported the use of proteomics to perform preliminary study on the type and amount of adsorbed proteins on the material surface [13–16], there was no study about the effect of adsorbed proteins on the material surface on subsequent cell adhesion and growth.

The interactions between biomaterials and proteins and between biomaterials and cells are two essential scientific issues of biocompatibility research. There is a close intrinsic connection among ‘material surface–protein adsorption’, ‘material surface–cell behavior’ and ‘protein adsorption–cell behavior’, which, however, have been studied individually by most researchers. Our previously study has explored a novel technical roadmap to combine cytological experiments, proteomic technology and bioinformatics analysis; and systematically investigated the molecular mechanism of mediation of adsorbed serum proteins to endothelial cells adhesion and growth on three biomaterials from an overall perspective of three aforementioned aspects [17].

In order to understand ‘why’ collagen/chitosan film has better cellular compatibility than chitosan, this article intends to undergo a comprehensive proteomics study on ‘material surface–protein adsorption–cell adhesion’ combining bioinformatics analysis. First, the differences of two materials were compared in the physicochemical properties and the cell adhesion and growth behavior. Subsequently, the proteomics and bioinformatics methods were performed to investigate the influence of hydrophilicity/hydrophobicity on the types and functions of adsorbed proteins on both surfaces. To identify more and new proteins that can mediate cell adhesion and growth on two surfaces, further analyses including the arg-gly-asp (RGD) and leu-asp-val (LDV) sequences of adsorbed proteins were carried out in addition to comparing the types and amounts of complex protein layers. Then, the different molecular mechanisms how serum adsorbed protein layers on two material surfaces mediated PC12 cell adhesion and growth were discussed and compared.

## Materials and methods

### Preparation of materials

#### Preparation of chitosan films

The chitosan powder with a deacetylation degree ≥85% (Jinan Heidebei Marine Bioengineering Co., Ltd., China) was dissolved in the 2% (v/v) acetic acid formulate the 1, 2 and 3% (w/v) chitosan solution, respectively. The chitosan films were formed on the surface of clean glass slides (8*8 mm^2^) via spin coating (3000 rpm, 3 min). After evaporation in a convention oven at 50°C for 12 h, the films were soaked into 1% (w/v) NaOH solution for 2, 4, 8 and 12 h, respectively, in order to neutralize the residual acetic acid. Then the films were rinsed with ultrapure water three times, and dried at room temperature.

#### Preparation of collagen/chitosan films

The 0.5 g acid-soluble Type I collagen (Chengdu Xinji Bioactive Collagen Development Co., Ltd) was dissolved in 25 ml of 0.5 M acetic acid to formulate the 2% (w/v) collagen solution. The 2% chitosan solution and the 2% collagen solution were mixed with a volume ratio of 4:6. The collagen/chitosan films were formed on the surface of clean glass slides via spin coating (3000 rpm, 3 min). After evaporation in a convention oven at 37°C for 12 h, the films were soaked in 1% (w/v) NaOH solution for 24 h, in order to neutralize the residual acetic acid. Then the films were rinsed with ultrapure water three times, and dried at room temperature.

The chitosan and the collagen/chitosan films were sterilized for 1 h each side by UV lamp before biological experiments.

### The characterization of morphology and composition of chitosan films

#### Scanning electron microscope

The surface morphology of the chitosan films with different chitosan concentration was characterized by Ultra Plus scanning electron microscope (SEM) (Zeiss, Germany).

#### Spectrum analysis

The chitosan films in different concentrations were analyzed using energy-dispersive X-ray spectroscopy, to detect the surface element composition.

### Characterization of chitosan and collagen/chitosan films

#### Contact angle measurement

The static contact angle measurements of the chitosan films (2% chitosan solution, the same in the remaining text) and the collagen/chitosan films were performed at room temperature using a goniometer (CAM200, KSV, Finland). A drop of double distilled water was placed on the surface of the dry film. A minimum of six measurements, taken at different positions on a film, were carried out.

#### Fourier transform infrared analysis

The IR spectra of chitosan and collagen/chitosan films were recorded in the 650-4000 cm^−1^ wavenumber range at a resolution of 4 cm^−^^1^ and 32-times by direct scanning under an Fourier transform infrared (FTIR) spectrometer with an OMNI-Sampler reflective attachment (Nicolet 5700, Thermo Fisher Scientific, USA).

### Cell culture

PC12 cells were cultured in the high-glucose medium supplemented with 10% (v/v) fetal bovine serum (Hangzhou Sijiqing Bioengineering Co., China) and 1% (v/v) penicillin-streptomycin (Gibco, USA); and were incubated in a cell incubator (Thermo Forma 3111, Thermo Fisher Scientific, USA) at 37°C under 5% CO_2_ and saturated humidity. The experiment was performed with cells in logarithmic growth phase.

### The observation of cell morphology

The chitosan films soaked in the NaOH solution for different time (2, 4, 8, 12 and 24 h) were placed in the 48-well plates. The PC12 cells were seeded on the surface of the materials with a cell density of 4.125 × 10^4^ cell/ml, 500 µl in each well. The cells were cultured for 24, 48 and 72 h, respectively. The medium was aspirated, and the cells were rinsed three times with phosphate buffer saline (PBS) and fixed for 10 min using 95% ethanol. The 0.02% acridine orange solution was added for staining under darkness for 15 min at 37°C. Then the cells were rinsed three times with PBS and the cell morphology was observed with Olympus BX51 fluorescence microscope (Olympus Corporation, Japan).

### Cell viability assay (methylthiazol tetrazolium assay)

The PC12 cells were cultured on the surface of chitosan and collagen/chitosan films for 24, 48 and 72 h, respectively; according to the experimental procedures described in the section ‘The observation of cell morphology’ The normal culture medium is served as the negative control, while the medium with 0.7% acrylamide solution is served as the positive control. At each time point, the methylthiazol tetrazolium (MTT) assay was performed to measure the cell proliferation rate in each experimental group [18].

### Measurement of dynamic curves of protein adsorption

The chitosan and the collagen/chitosan films were placed in 60-mm diameter tissue culture polystyrene dishes (Corning Inc., USA), respectively. A 5 ml high-glucose DMEM medium supplemented with 10% fetal bovine serum was added to each dish, and incubated at 37°C under 5% CO_2_ for 1, 2, 3, 4 and 5 h. For each material three parallel samples were prepared at each time point. After incubation, the medium was aspirated and the dishes were rinsed once with PBS. A 500 μl buffer solution (8 M Urea, 0.1 M TrisBase, 0.01 M DTT, pH 8.6) was pooled to the surface of the materials and incubated for 20 minutes, and the adsorbed proteins on the surface of the materials were collected afterwards. Another 500 μl protein eluent was pooled to repeat the collection procedure once more. Then the dishes were rinsed once with PBS. All of the wash solutions were collected, respectively. The protein content in the wash solutions was determined by using the Bradford method [19].

### MS identification of adsorbed serum proteins

According to the experimental procedures described in the section ‘Measurement of dynamic curves of protein adsorption’, the chitosan and the collagen/chitosan films were incubated in the culture medium supplemented with 10% bovine serum at 37°C for 4 h. The adsorbed proteins were eluted, collected, dialyzed and then lyophilized separately, and the lyophilize powder was collected. The 1D sodium dodecyl sulfate polyacrylamide gel electrophoresis, the MS identification and the calculation of protein content were conducted by ProtTech Inc., USA. The procedures were listed as follows: The gel was cut into 16 bands. Each band was digested by trypsin, and the MS data were collected using LC-MS and LC-MS/MS. ProtTech’s ProtQuest software was used to perform the latest non-redundant search, and the searched results were analyzed and verified manually. Finally, the relative content of each protein in the samples was calculated using the label-free protein quantification method.

### Bioinformatics analysis

The biological pathway analysis of the identified adsorbed proteins was performed by DAVID (http://david.abcc.ncifcrf.gov/). The functional motif sequence analysis of the proteins identified in proteomics was performed by ScanProsite (http://Prosite.expasy.org/scanprosite/).

## Results and discussion

### Composition and morphology of chitosan film

**Figure 1.**
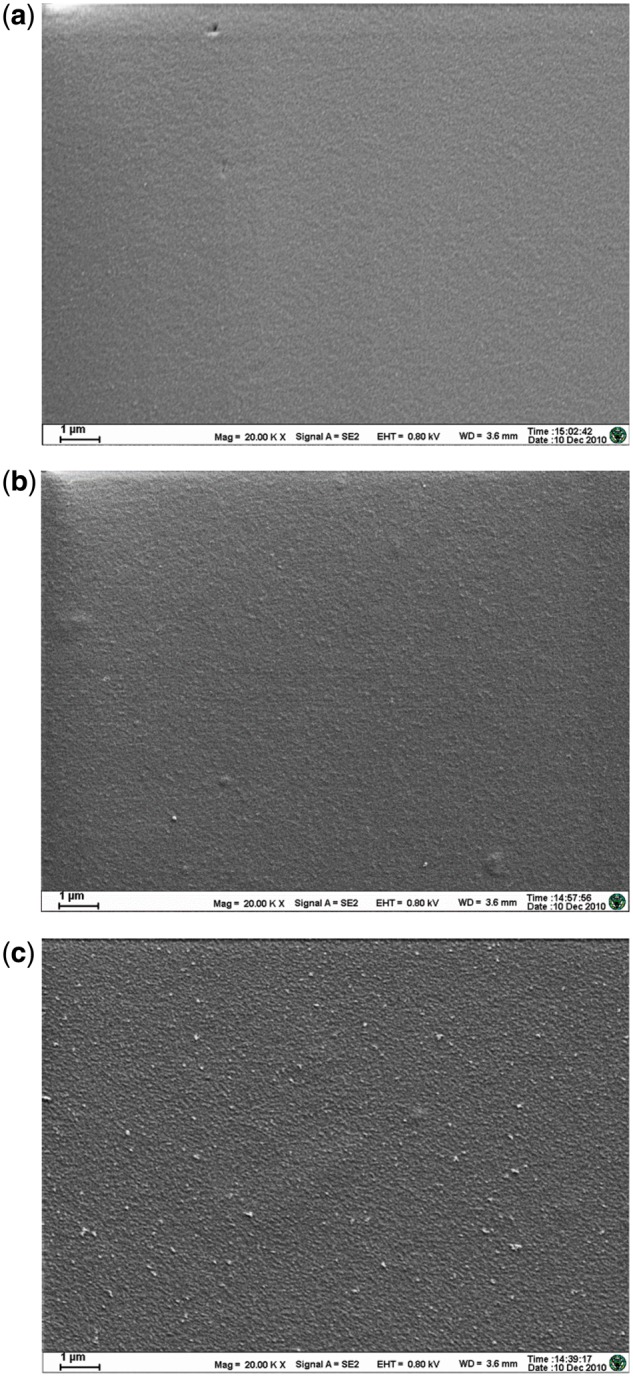
The SEM of chitosan films in different concentrations, **(a)** 1%, **(b)** 2% and **(c)** 3%

The chitosan films of different concentrations were characterized by the SEM and the X-ray energy-dispersive spectroscopy (EDS) (Fig. 1 and Table 1). The surfaces of the chitosan films of 1 and 2% concentrations of were relatively smooth whereas the film of 3% chitosan solution was relatively rough with many tiny granular protrusions. The EDS map revealed a small amount of Si in the 1% chitosan surface and that the composition of C, N and O was quite different from those on the of 2 and 3% chitosan surfaces. This indicated that the film prepared with the 1% chitosan solution was too thin to completely cover the glass substrate and thus the Si element in the glass was detected. Therefore, the 1% chitosan film was not a suitable material for subsequent cell culturing and protein adsorption experiment. On the surfaces of the 2 and 3% chitosan films, only C, N and O elements were revealed in the EDS mapping with an atomic ratio between C and N close to 3: 1. The agreement with the composition of chitosan molecule indicated that the glass substrate was completely covered.

**Table 1.** The surface element composition of chitosan films in different concentrations

Chitosan concentration (w/v)	Element							
	Weight (%)				Atom (%)			
	C	N	O	Si	C	N	O	Si
1%	29.66	9.25	60.02	1.06	35.69	9.55	54.22	0.55
2%	39.26	15.34	45.40		45.39	15.21	39.40	
3%	40.43	16.74	42.83		46.50	16.51	36.98	

In addition, the 3% chitosan solution had large viscosity and thus was difficult handling. The 2% chitosan solution had a moderate viscosity allowing easier handling, and resulted in films with smoother surface and better reproducibility. Given the above advantages, the chitosan film was prepared with the 2% chitosan solution for the subsequent cell experiment and protein adsorption experiment.

### Characterization of chitosan and collagen/chitosan films

#### Results of contact angle measurement

Hydrophobicity or hydrophilicity, as an important physical and chemical property of the material surfaces, affects *in vitro* biological behaviors such as cell adhesion, migration, proliferation, and differentiation [20]. The hydrophilic material surfaces are more favorable to penetration of the medium as well as cell adhesion and growth due to the cell film’s hydrophilicity [21]. The contact angle θ, as an indicator of the surface wettability, can be used to characterize the hydrophobicity of the material. Some researches defined the boundary between hydrophilicity and hydrophobicity as a water contact angle of 65° [22, 23]. The contact angle of the chitosan film was measured as 86° ± 2°, showing hydrophobicity. Whereas the collagen/chitosan film had measured contact angle of 64° ± 2°, which was significantly smaller (*P* < 0.05) than that of the chitosan film and demonstrates hydrophilicity.

#### Results of IR spectroscopy

**Figure 2.**
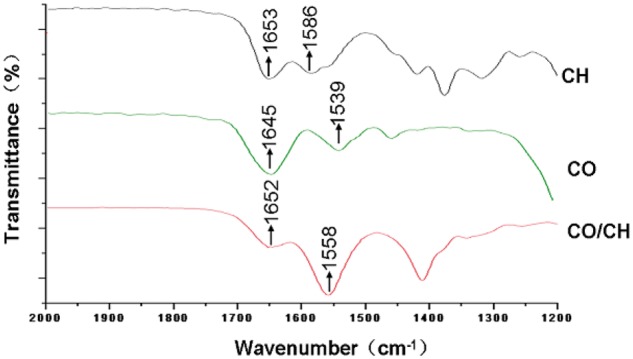
IR spectra of chitosan film (CH), collagen (CO), and collagen/chitosan mixture film (CO/CH)

The IR spectroscopy results of both films are shown in Fig. 2 (spectra between 1200 and 2000 cm^−1^). The spectrum of the chitosan film exhibited an acetyl amide I band at 1653 cm^−1^ and an absorption peak of the characteristic amino group at 1586 cm^−1^. The spectrum of the pure collagen exhibited an absorption peak of the characteristic carboxyl group at 1645 cm^−1^ and a peak of the amino group at 1539 cm^−1^ [24]. Incorporation of collagen led to the shifts of the amide I band and the peak of amino group in the spectrum of chitosan, implying the formation of hydrogen bonds between the chitosan and collagen molecules in agreement with the previous studies [24, 25].

### Morphology of PC12 cells on the chitosan surface after soaking in NaOH solution for different time

**Figure 3.**
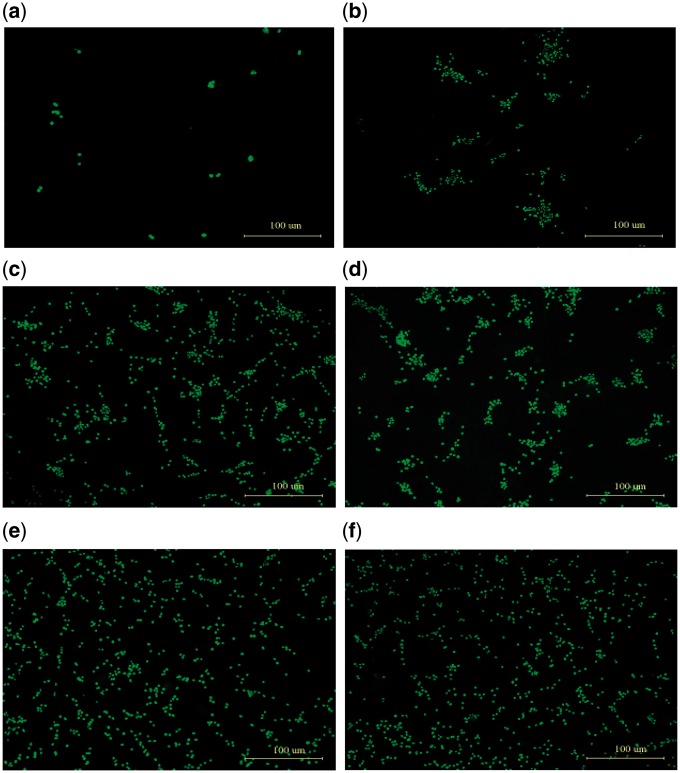
The fluorescence microscopy images of the PC12 cells cultured on chitosan films treated with NaOH solution for different time. **(a)** 0 h, **(b)** 2 h, **(c)** 4 h, **(d)** 8 h, **(e)** 12 h and **(f)** 24 h. Green fluorescence shows cell nuclei

Since the use of acetic acid to dissolve chitosan powder might result in residual H^+^ on the chitosan film and thus might affect cell adhesion, we followed the procedures in some previous studies [26, 27] and soaked the chitosan films in the 1% NaOH solution for 2, 4, 8, 12 and 24 h, respectively. The fluorescence microscopy of PC12 cells grown on the surface of chitosan film for 48 h are shown in Fig. 3, with panels (a) to (f) showing films after 0 h, 2 h, 4 h, 8 h, 12 h, and 24 h of soaking, respectively. The cells hardly adhered to the surface of the chitosan film before soaking in the NaOH solution (Fig. 3a), indicating that the residual H^+^ interfered with PC12 cell adhesion. Cell adhesion increased with the film’s soaking time in NaOH with cell clusters present on the films with 2 h, 4 h, and 8 h NaOH soaking (Fig. 3b–d). Fewer clusters were present on the film with more than 12-h soaking (Fig. 3e); and the clusters disappeared on the film soaked for 24 h (Fig. 3f), indicating very good cell adhesion. This confirmed that the use of NaOH solution had effectively neutralized the residual H^+^ on the chitosan film; and therefore, the film soaked in the 1% NaOH solution for 24 h were used in the following experiments.

### Cell proliferation rate (MTT assay)

**Figure 4.**
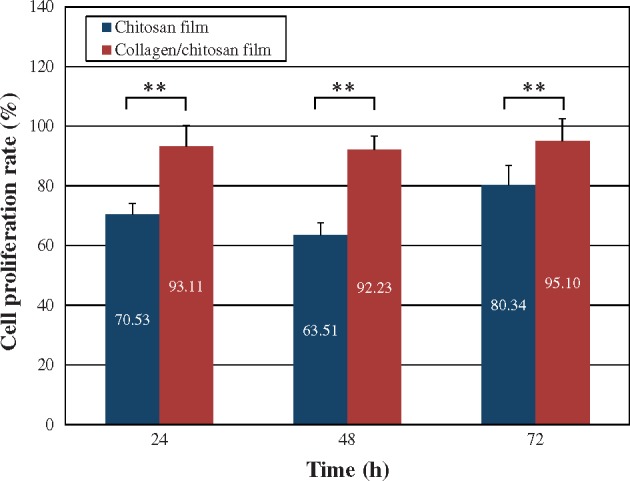
The cell proliferation rate of the PC12 cells cultured on both surfaces changed at different time points. Results were expressed as mean ± SD (*n* = 6). **P < 0.01

The PC12 cells cultured on both surfaces (Fig. 4) had the proliferation rate significantly higher on the collagen/chitosan film compared with the chitosan film (*P* < 0.01) for all three culture durations (24, 48 and 72 h). The MTT assay showed the results in agreement with those of previous studies [25, 28] that the collagen/chitosan film significantly promoted the PC12 cell growth and proliferation compared with the pure chitosan film (Fig. 4).

### Dynamic curves of protein adsorption

**Figure 5.**
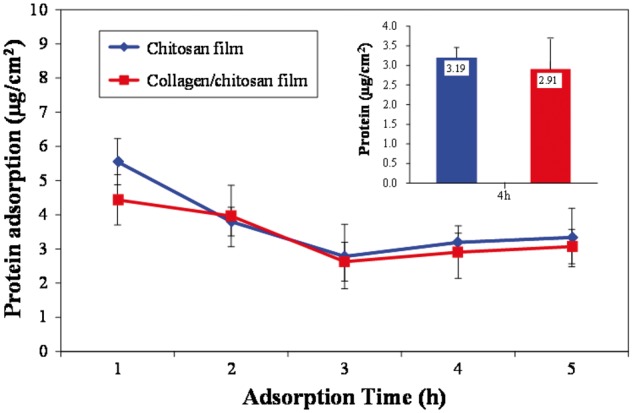
The protein adsorption on both surfaces for different incubation time in the DMEM medium containing 10% fetal bovine serum. Each data are presented as mean ± SD (*n* = 3). There was no significant difference at each time point (*P* > 0.05)

Figure 5 shows the comparison about the kinetics of protein adsorption on both surfaces. In general, the temporal profile of protein adsorption was consistent on both surfaces: the maximum adsorption was obtained at 1-h incubation while the minimum was obtained at 3-h incubation; the adsorption increased slightly after incubating for 4 h and then reached a balance afterwards. Although the total protein adsorption on the chitosan surface was slightly more than that on the collagen/chitosan surface, there was no significant at each time point (*P* > 0.05). At 4 h, the total adsorption on the both surfaces was 3.19 and 2.91 μg/cm^2^, respectively.

Protein adsorption is an intricate process influenced by many factors such as hydrophobic effect, electrostatic force, and hydrogen bonding and van der Waal’s interaction, among which hydrophobic effect and electrostatic force play the most important role in affecting the interactions between proteins and materials [29]. Studies have demonstrated that the hydrophobic surfaces are more protein-adsorbent because of the strong hydrophobic interactions occurring between the hydrophobic surface and the hydrophobic structure of the protein [30]. According to the results of contact angle measurement, the contact angle of chitosan surface (86.46°) is significantly greater than that of the collagen/chitosan surface (64.00°) (*P* < 0.05) and more hydrophobic. Yet in agreement with their hydrophobic property, the results of dynamic curves showed that the total protein adsorption on the former was slightly more than that on the latter, there was no significant at each time point (*P* > 0.05). At 4 h, the total adsorption on two surfaces remained stable, indicating that the adsorption had reached a kinetic equilibrium. Thus, 4 h was chosen as the incubation time for the subsequent experiment.

### Results of proteomics

Proteomics analysis indicates that 104 serum proteins were adsorbed on the chitosan surface and 98 on the collagen/chitosan surface, respectively; 78 were adsorbed on both surfaces. In total 26 proteins were differentially adsorbed on the chitosan surface whereas 20 were differentially adsorbed on the collagen/chitosan surface (Table 2).

**Table 2.** The kinds of adsorbed proteins on chitosan and collagen/chitosan surfaces

Material	Kinds of adsorbed proteins		
	Total number	Adsorbed on both surfaces	Differential adsorption
Chitosan film	104	78	26
Collagen/chitosan film	98		20

More detailed information of protein adsorption is provided in the Supplementary Tables S1–S3. The differential adsorption on the chitosan surface was 2.756% of the total adsorption (Supplementary Table S2); whereas the differential adsorption on the collagen/chitosan surface was 0.678% of the total (Supplementary Table S3).

### Bioinformatics analysis

#### Analysis of biological pathways

**Figure 6.**
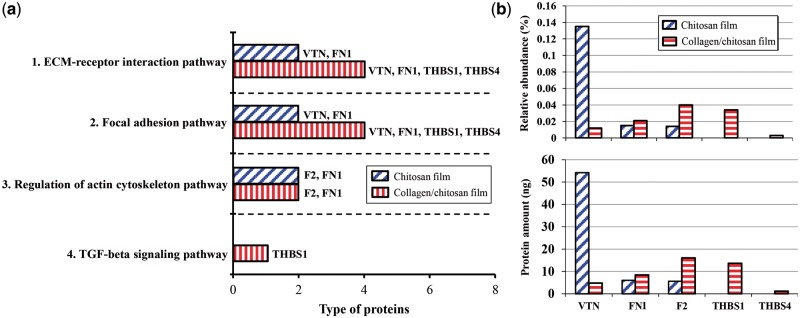
(a) Four biological pathways contributed by adsorbed proteins for cell adhesion and growth on the chitosan and collagen/chitosan surfaces. (B) Five adsorbed proteins and their adsorption in four pathways on the both surfaces. The amount of protein = relative abundance × total protein adsorption. The total amount of protein adsorbed on both surfaces was 40.13 and 36.52 μg, respectively

The results of biological pathway analysis indicate 40 biological pathways involved in the protein adsorption on the chitosan surface and 19 on the collagen/chitosan surface; 16 pathways were activated on both surfaces (Supplementary Table S4). The adsorbed proteins as ligand-receptor binding contributed to nine pathways (Supplementary Table S5). Four pathways were closely related to the interactions between cell and extracellular proteins for cell adhesion and growth (Fig. 6). Figure 6a listed 5 adsorbed proteins that activated these four pathways: vitronectin (VTN), fibronectin 1 (FN1), thrombospondin 1 (THBS1), thrombospondin 4 (THBS4) and coagulation factor II (F2). Figure 6b illustrated their adsorption, four of which–FN1, F2, THBS1 and THBS4–on the collagen/chitosan surface were significantly higher than that on the chitosan surface.

**Figure 7.**
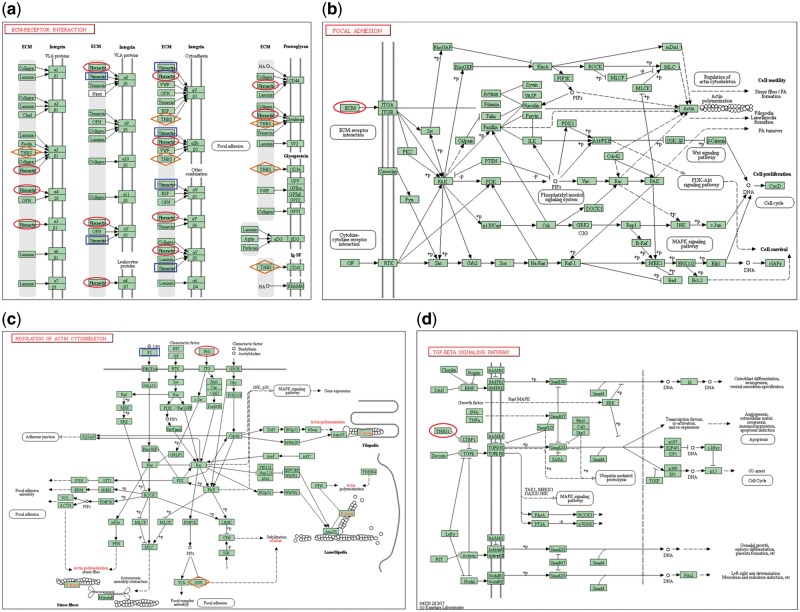
(a) ECM-receptor interaction pathway, **(b)** focal adhesion pathway, **(c)** regulation of actin cytoskeleton pathway, **(d)** TGF-β signaling pathway [31]

Cell adhesion is accomplished by the cell adhesion molecules located on the cell surface such as integrins, immunoglobulin superfamily, cadherins and selectins. Binding of the adsorbed proteins to the receptors on the surface activates cell signaling and cell surface integrins, which then trigger a series of signaling pathways and promote cell adhesion and proliferation [17]. Figure 7a and b illustrate that the ECM-receptor interaction pathway and focal adhesion pathway were activated by two adsorbed proteins (VTN, FN1) on the chitosan surface and four proteins (VTN, FN1, THBS1 and THBS4) on the collagen/chitosan surface, respectively. Figure 7c shows that the regulation of actin cytoskeleton pathway was activated by two proteins (F2, FN1) on both surfaces. Figure 7d shows that the transforming growth factor-beta (TGF-β) signaling pathway was activated by the differentially adsorbed protein (THBS1) on the collagen/chitosan surface.

The ECM is a reticular structure composed of secretory proteins and polysaccharides. It is of vital importance in morphogenesis and in providing structural and functional support for tissues and organs. One class of extracellular macromolecules is adhesive glycoproteins including VTN, FN1, THBS, THBS4 etc. These glycoproteins bind to such ECM structural molecules as collagen and proteoglycans, and on the other hand, connect to cell surface receptors, thereby anchoring the cells to the ECM. The interaction between cells and ECM directly or indirectly controls cell adhesion, migration, differentiation, proliferation, and apoptosis. Integrins are the main receptors that mediate cell’s anchoring to the ECM. VTN domain contains not only the RGD integrin-binding motif which can be recognized by six cell surface integrins (α_8_β_1_, α_v_β_1_, α_v_β_3_, α_IIb_β_3_, α_v_β_5_ and α_v_β_8_), but also other binding sites to connect collagen, heparin, complement components, perforin, plasma zymogen, and more [32]. FN1, a high molecular weight glycoprotein, is a critical component of ECM and plays a vital role in the biological processes such as cell adhesion, migration, growth, and differentiation. Each subunit of FN1 consists of several domains, and is able to interact with 10 integrins (α_3_β_1_, α_4_β_1_, α_5_β_1_, α_8_β_1_, α_v_β_1_, α_4_β_7_, α_v_β_3_, α_IIb_β_3_, α_v_β_6_ and α_v_β_8_) and 2 proteoglycans (CD44, Syndecan) on the cell film surface. THBS1 and THBS4, members of the thrombospondin family, are able to bind to 3 integrins (α_3_β_1_, α_v_β_3_ and α_IIb_β_3_) and 3 proteoglycans (Syndecan, CD36 and CD47). Therefore, it reveals that the above four proteins mediate a series of reactions between cells and ECM and promote cell adhesion via binding to a variety of integrins.

Focal adhesion is the connection between ECM and actin fibers. There are two types of signaling pathways are mediated by focal adhesions. The first one is the interaction between the ECM components and the integrins, which activates the tyrosine kinases (Src); the activated Src phosphorylates tyrosine residue of focal adhesion kinase and further promotes downstream signaling, thereby regulating cell growth and proliferation. The second one is the interaction between cytokines and cell surface cytokine receptors which results in converting extracellular signals into intracellular signals. As the components of ECM, VTN, FN1, THBS1 and THBS4 all interact with cell surface integrins (integrin α subunits, integrin β subunits) via the first type of pathways, causing the activation of Src and the downstream focal adhesion pathway. In this article, the collagen/chitosan surface adsorbed more FN1 and differentially adsorbed THBS1 and THBS4; whereas the chitosan surface adsorbed more VTN. Though the studies defined FN1 and VTN adsorption to promote cell adhesion [33, 34], FN1 was identified stronger adhesive capability other than VTN [33]. Therefore, four proteins on the collagen/chitosan surface provided stronger activation of ECM-receptor interaction pathway and focal adhesion pathway than those on the chitosan surface.

Actin is a critical component of cytoskeletal microfilaments and has two forms at a homeostatic balance in cells in response to extracellular stimuli: monomeric globules called G-actin and polymeric filaments called F-actin [35]. The actin cytoskeleton mediates a variety of essential biological functions in all eukaryotic cells. In addition to providing a structural framework around which cell shape and polarity are defined, its dynamic properties provide the driving force for cells to move, to divide, to differentiate and to phagocytose [36, 37]. F2 is a serine protease. Additional evidence was supported by an *in vivo* study that F2 could affect cell morphology and gap formation between cells, mainly through the reassembly of actin filaments [38]. By binding to the cell surface receptor F2R, F2 transmits extracellular signals into activation of intracellular pathways, leading to the enhancement of actin stress fiber formation and the regulation of actin cytoskeleton assembly [39]. The binding of FN1 to integrins not only induces cell adhesion but also enhances the actin stress fiber formation [40]. In this article, more F2 and FN1 were adsorbed on the collagen/chitosan surface than on the chitosan surface, therefore, provided stronger activation of regulation of actin cytoskeleton pathway.

The TGF-β signaling pathway is a signal transduction pathway resulting from the binding of cell surface TGF-β receptors (e.g. TGFBR1 and TGFBR2) to extracellular ligands. THBS1 allows TGF-β to bind its receptors (TGFBR1 and TGFBR2) making for the activation of TGF-β signaling pathway [41]. The activated TGF-β signaling pathway can control a diverse set of cellular processes (including cell proliferation, recognition, differentiation, apoptosis, etc.) through transcriptional regulation of target gene expression [42, 43]. In this article, THBS1 was only adsorbed on the collagen/chitosan surface and TGF-β signaling pathway could be activated, while the TGF-β signaling pathway could not be activated on the chitosan surface because no THBS1 was adsorbed on it.

The adsorption of five proteins involved in four pathways for cell adhesion and growth are shown in Table 3. Three identical proteins (VTN, FN1 and F2) were adsorbed on both surfaces. The adsorption of VTN on the chitosan surface (0.135%) was greater than that on the collagen/chitosan surface (0.012%); whereas the adsorption of FN1 and F2 on the latter (0.021 and 0.040%) were greater than that on the former (0.015 and 0.014%, respectively). Table 3 further indicates that two adhesive glycoproteins, THBS1 and THBS4, were differentially adsorbed on the collagen/chitosan surface, and accounted for 0.034 and 0.003% of the protein adsorption, respectively. In addition to binding to three integrins and three proteoglycans on the cell film, THBS1 and THBS4 can bind to fibrinogen, fibronectin, laminin and collagen V, potentially facilitating cell adhesion, cytoskeleton assembly and expression of related genes; and further drive a series of biological processes such as morphogenesis, chemotaxis, axon formation and cell cycle progression [17, 44, 45].

**Table 3.** The adsorption of five proteins involved in cell adhesion and growth on the chitosan and collagen/chitosan surfaces

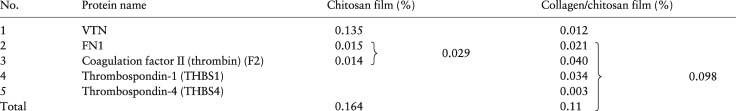

Overall, the total adsorption of three proteins adsorbed on the chitosan surface (0.164%) was greater than that of the 5 proteins (0.11%) on the collagen/chitosan surface, but mainly due to the relatively large adsorption of VTN on the chitosan surface. VTN and FN1 are both able to bind to cell surface receptors and induce corresponding biological processes. However, FN1 contains twice as many binding sites that bind to integrins (12 sites) as VTN (6 sites) (Fig. 7a), also FN1 contains polypeptide sequences such as CS-1 [46]. Therefore, FN1 has stronger adhesive capability than VTN [33]. As VTN not calculated in Table 3, the protein adsorption on the collagen/chitosan surface (0.098%) was 3.38 times more than that on the chitosan surface (0.029%). Thus collagen/chitosan surface was in favor of promoting cell adhesion and growth than chitosan surface.

#### Results of protein function analysis

The functions of a protein are determined by its sequence, and proteins with similar sequences have similar functions. Some relatively short conserved sequences are typically related to specific biological functions by serving as the functional sites such as ligand binding sites, catalytic sites of enzymes etc. This article aims to further analyze the RGD and LDV sequences of all the identified adsorbed proteins, in order to discover more proteins that might be associated with cell adhesion and growth and thus enable a more comprehensive analysis of the role of adsorbed proteins in mediating the response of cells to biomaterials.

##### Results and analysis of RGD sequence

The RGD sequence (RGD) is an ‘arginyl (R)-glycyl (G)-aspartic acid (D)’ tripeptide sequence existing in diverse ECM protein structures. Since the RGD motif promotes cell adhesion through binding of various cell surface integrins, it is a functional protein sequence that plays a major role in cell adhesion. Table 4 shows the information of adsorbed protein containing RGD on both surfaces. Among eight adsorbed proteins containing RGD on two surfaces altogether, six proteins that were adsorbed on both surfaces were VTN, serine family F member 2 (SERPINF2), complement factor H (CFH), FN1, F2 and fibulin-1 (FBLN1). One protein differentially adsorbed on the chitosan surface was insulin-like growth factor I (IGF1); one protein differentially adsorbed on the collagen/chitosan surface was THBS1.

**Table 4.** The results of RGD sequence in the adsorbed proteins on the chitosan and collagen/chitosan surfaces

No.	Gene ID	UniProt	Protein name	Location of RGD sequence in protein domain	Relative abundance (%)	
					Chitosan film	Collagen/chitosan film
1	507525	Q3ZBS7	VTN	64–66	0.135	0.012
				128–130		
2	282522	P28800	SERPINF2	466–468	0.037	0.048
3	280816	Q28085	CFH	246–248	0.019	0.029
4	280749	P07589	FN1	1616–1618	0.015	0.021
				2183–2185		
5	280685	P00735	Coagulation factor II (F2)	563–565	0.014	0.040
6	514588	A5D7S8	FBLN1	95–97	0.004	0.003
7	281239	P07455	IGF1	250–252	0.051	
8	282530	Q28178	THBS1	926–928		0.034
Total					0.275	0.187

In Table 4, four adsorbed proteins (VTN, FN1, F2 and THBS1) were found participated in the pathways for cell adhesion and growth (Fig. 6), while another four proteins (SERPINF2, CFH, FBLN1 and IGF1) were newly found in RGD sequence search. SERPINF2 is a member of the serpin family (serine protease inhibitor), the main physiological inhibitor of plasmin, and a key regulator of fibrinolytic system. Thomas et al. [47] studied the interaction between the endothelial cells and the C-terminus of α2AP (SERPINF2). The result demonstrated that the interaction depended largely on the RDG sequence with most but not all of the binding being integrin-mediated, indicating that α2AP might potentially play a role in the control of regulating cell functions. CFH protein family includes factor H, factor H-like protein 1 and factor H-related protein 1–4, the most representative of which is factor H. Factor H is a multidomain and multifunctional plasma protein and has multiple physiological activities: it acts as an ECM component; binds to cellular receptors of the integrins; and interacts with a wide selection of C-reactive protein, thrombospondin, bone sialoprotein, osteopontin, and more [48]. FBLN1 is an adhesion modulatory protein which binds to the C-terminal heparin-binding (HepII) domain of FN and prevents HepII binding to the cell surface receptor, syndecan-4. This causes reduced cell interactions with adhesive ECM, thereby to impede cell adhesion, spreading, cell movement, morphogenesis, proliferation, and other related processes [49]. IGF1 is a member of the insulin family with high similarity to insulin both structurally and functionally; it is a mediator of growth hormone and is able to promote growth [50].

Seven proteins containing RDG were adsorbed on chitosan and the collagen/chitosan surfaces, respectively. Although a greater total amount was found on the chitosan surface (0.275%) compared with the collagen/chitosan surface (0.187%), the amount of five proteins containing RGD (SERPINF2, F1, CFH, F2 and THBS1) were greater on the latter. The chitosan surface only had three proteins containing RGD (VTN, FBLN1 and IGF1) with greater adsorption.

##### Analysis of LDV sequence

The LDV sequence (leucine (L)-aspartic acid (D)-valine (V), LDV) was first discovered in the fibronectin CS-1 sequence and is the most prevalent recognition sequence of integrins besides RDG. LDV is functionally relevant to RGD [51] and is able to bind to integrins α_4_β_1_, α_4_β_7_ and α_9_β_1_. When compared with the RGD motif, the LDV motif has a higher specificity for binding to the integrins, mainly binding to α_4_β_1_ [52].

In Table 5, 12 adsorbed proteins were found containing LDV. Among them, 2 proteins (FN1 and THBS1) were found participated in the pathways for cell adhesion and growth (Fig. 6), while another 10 proteins were newly found in LDV sequence search. A total of 10 proteins containing LDV were adsorbed on the chitosan surface whereas 8 were adsorbed on the collagen/chitosan surface; 6 proteins were adsorbed on both surfaces (Table 5). In terms of adsorption, the proteins containing LDV accounted for 5.443 and 6.656% of the total adsorption on the chitosan and the collagen/chitosan surfaces, respectively, and their role in mediating cell behavior cannot be neglected. Among six proteins adsorbed on both surfaces, five had greater amount on the collagen/chitosan surface, with the serine protease inhibitor A3-1 being the only exception.

**Table 5.** The analysis of LDV sequence

No.	Gene ID	UniProt	Protein name	Location of LDV sequence in protein domain	Relative abundance (%)	
					Chitosan film	Collagen/chitosan film
1	497200	Q3SZR3	Orosomucoid 1	161–163	3.919	4.792
2	286804	Q9TTE1	Serine A3-1 (SERPINA3-1)	355–357	0.797	0.716
3	280677	Q2UVX4	Complement component C3	1293–1295	0.301	0.341
4	504615	Q58D62	Fetuin-B	87–89	0.271	0.535
				344–346		
5	497203	Q3SZQ8	SERPINA3-7	196–198	0.028	0.078
				359–361		
6	280749	P07589	FN1	2103–2105	0.015	0.021
				2467–2469		
7	282535	O19334	Major histocompatibility complex, class II, DQ alpha 2 (Bos taurus major histocompatibility complex, class II-DQA2)	96–98	0.093	
8	533307	Q2KJD0	Tubulin beta Class I	117–119	0.010	
9	280820	Q9BGU1	Histidine-rich glycoprotein	74–76	0.007	
10	5852893	A8QEP3	Hypothetical protein (MGL_4276)	94–96	0.002	
11	100272170	A2I7M9	SERPINA3-2	355–357		0.139
12	282530	Q28178	THBS1	179–181		0.034
Total					5.443	6.656

In summary, although the collagen/chitosan surface (0.187%) had less adsorption of all proteins containing RGD than the chitosan surface (0.275%), it had greater adsorption for five of the individual proteins. On the other hand, the collagen/chitosan surface (6.656%) not only had greater adsorption of all proteins containing LDV than that on the chitosan surface (5.443%), it also had greater individual adsorption for seven of the proteins.

**Figure 8.**
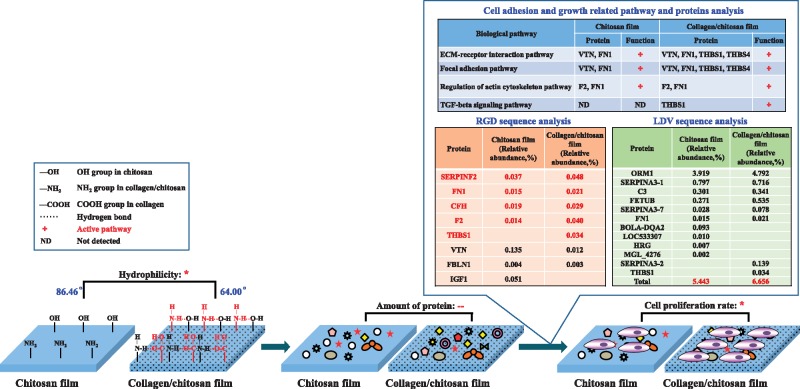
The impact of adsorbed proteins on PC12 cell adhesion and proliferation between the chitosan and the collagen/chitosan films. *represents significant difference (*P* < 0.05); –represents no significant difference (*P* > 0.05)

It has been reported that surface functional groups have significant effects on protein adsorption and cell behaviors [53]. In the collagen and chitosan mixture, the –OH groups of hydroxyproline and the –COOH and NH_2_ end groups in collagen can form hydrogen bonds with –OH and NH_2_ groups in chitosan, which alters the helical structure of collagen [7, 24] and the mechanical properties of the material, and eventually influences cell adhesion [54]. Figure 8 compares of the impact on protein adsorption and PC12 cell proliferation between the chitosan and the collagen/chitosan films. The chitosan film by itself had hydrophobicity, but the collagen/chitosan mixture film had relatively good hydrophilicity with the hydrogen bonds formed after adding collagen; as hydrophilic surfaces were more conducive to cell adhesion and growth, the significant difference (*P* < 0.05) of PC12 cell proliferation rate on both surfaces was consistent with the significant difference (*P* < 0.05) of their hydrophilicity or hydrophobicity. It has been well-accepted in recent years that cell adhesion on material surface is mediated by proteins, and a surface with appropriate hydrophilic-hydrophobic balance is conducive to protein adsorption whereas a very hydrophilic surface is not. The contact angle of the collagen/chitosan film (64°) is close to the well-recognized 65° contact angle for hydrophilic-hydrophobic balance. The collagen/chitosan surface adsorbed more kinds and quantities of proteins and activated more pathways that facilitate cell adhesion and growth than the chitosan surface. Furthermore, the collagen/chitosan surface adsorbed more kinds of proteins containing RGD as well as more quantities of proteins containing LDV than the chitosan surface. Therefore, the collagen/chitosan film was more conducive to promoting cell adhesion and growth.

It is known, protein adsorption on the biomaterials surface is an intricate process influenced by many factors including the properties of material such as physicochemical property, hydrophobicity, morphology, mechanical property, and electrical property and the properties of protein such as concentration, molecular size, chargeability, and conformation etc. In order to understand why certain proteins adsorb differently on different surfaces, many methods have been used to investigate the adsorption behavior on different surfaces. Polyethylene oxide (PEO) can be used to build an anti-protein adhesion surface, which is likely related to the flexibility and hydrophilicity of PEO chains giving a high excluded volume [55]. Besides, the adsorption of plasma proteins on the phosphatidylcholine and other neutral (or charge-shielded) phospholipid surfaces is relatively low, and similar to that on the PEO surface [56, 57]. Feng et al. [58] used poly(2-methacryloyloxyethyl phosphorylcholine) (poly(MPC)) brushes over different graft density and chain length to study the Fib adsorption on the silicon wafer surfaces. The results implied that the Fib adsorption was determined by both graft density and chain length though; it showed a stronger dependence on graft density than on chain length. Unsworth et al. [59] investigated the effects of PEO chain density, chain length, and end-group on protein adsorption, and suggested that chain density might be the key property for suppression of Fib adsorption. Zhao et al. [60] used microplate reader to detect the exposure degree of C terminal function area of fibrinogen (Fib) γ-chain (recognized by the platelet Glycoprotein IIb/IIIa receptor) and to quantify Fib degeneration on the TiO_2_ films with vacuum thermal treatment. The results proved that the hydrophilic TiO_2_ could significantly decrease Fib denaturation. Fan et al. [61] used quartz crystal microbalance to determine the adsorption of bovine serum albumin (BSA) and Fib on the L- and D-selenocystine (L-Se/D-Se) immobilized TiO_2_ films, and found that surface chirality led to different steric interactions between the negatively charged L-Se/D-Se surfaces and the negatively charged BSA and Fib, which in turn influenced hydrogen bonding and hydrophobic interaction, and eventually influenced protein adsorption. Svendsen et al. [62] used ellipsometry to study the competitive adsorption of human serum albumin (HSA), human immunoglobulin G (IgG) and laminin-1 on the spin-coated and sintered hydroxyapatite (HA) surfaces. The results showed that the differences in surface roughness and chemical composition (the spin-coated HA surface contained titanium and aluminum, whereas the sintered surface contained sodium) between two types of HA substratum contributed to different adsorption properties of proteins.

Our team previously has used biosensors, atomic force microscopy, IR spectroscopy, UV/Visible spectrophotometry, weighing method and molecular dynamics simulation to study the adsorption properties of single proteins on different materials and the competitive adsorption of three proteins [10–12, 63–65]. When compared with polystyrene, the special morphology, surface energy and interfacial energy of polyurethane (PU) surface had a significant effect on the types and amounts of adsorbed proteins [12], and its hydrophobic property was closely related to the change of protein conformation [63]. More BSA, Fib and IgG were adsorbed on HA than H50-50 PU [64]; the former was conducive to promoting blood coagulation and bone repair. Studies on adsorption behaviors of HSA, Fib and IgG on polymethylmethacrylate (PMMA) and its three coatings (DLC, CN_0.088_ and CN_0.15_) showed that the amounts of adsorbed proteins on four surfaces (DLC>CN_0.088_>PMMA >CN_0.15_) was in the same order as the hydrophobicity of four surfaces [10].

Since the aforementioned classical methods have addressed the question why certain proteins adsorb differently on different surfaces from a single perspective, they are limited to study the adsorption behavior of a single or a few known proteins on the material surface. However, biomaterials in actual use come into contact with a complex protein environment (e.g. blood) with a wide variety of proteins, whose adsorption behaviors are very complicated. Therefore, the high-throughput proteomics and bioinformatics techniques are required for studying the complex adsorbed protein layers on the material surface. With advantages of high-throughput and being exhaustive, using proteomic tools to screen adsorbed proteins not only obtain information about the types and amounts of adsorbed proteins but also identify new proteins that affect cell function, thus enabling us to further explore how various adsorbed proteins affect cell adhesion and growth. On the other hand, the high-throughput information provided by proteomics and bioinformatics is unable to answer the question why certain proteins adsorb differently on different surfaces simultaneously. Therefore, the author proposed that the high-throughput proteomic research should be combined with targeted classical research. The proteomic approach provides the information (e.g. the amounts, types, functions etc.) of the exhaustive adsorbed protein layer on the material surface; the proteomic analysis screens out important proteins and/or newly identified proteins; and the classical methods are used to verify to the adsorption behavior of these important and/or newly identified proteins. So, it is not only helpful to understand how different type, amount and function of adsorbed proteins on the material surface effect subsequent cell adhesion and growth, and the question why certain protein among protein groups adsorb differently on different surfaces can also be answered.

## Conclusion

No other literature but this article has combined proteomic technology with bioinformatics analysis to compare the components and functions of adsorbed protein layer on the chitosan and collagen/chitosan surfaces. Although the results showed no significant difference in the total amount of adsorbed proteins on both surfaces, the collagen/chitosan film had significantly better hydrophilicity with the hydrogen bonds formed after adding the more biocompatible collagen and thereby, compared with the pure chitosan film, adsorbed different types and functions of proteins. The collagen/chitosan surface adsorbed more FN1, THBS1, THBS4 and F2, which were more conducive to activating ECM-receptor interaction pathway, focal adhesion pathway, regulation of actin cytoskeleton pathway, and TGF-β signaling pathway. In addition, the collagen/chitosan surface adsorbed more kinds of proteins containing RGD and more quantities of proteins containing LDV than the chitosan surface. Therefore, the collagen/chitosan film was more conducive to promoting cell adhesion and growth. This study showed that the surface functional groups caused a significant distinction in their hydrophilicity and hydrophobicity, which thereby led to different types and functions of adsorbed proteins, and eventually influenced cell adhesion and growth on two surfaces. The overall analysis of ‘material–protein–cell behavior’ in this article revealed the molecular mechanisms of different cellular compatibility of two materials.

## Supplementary Material

Supplementary Information
