# Supplementary material for: A proteomics study to explore the role of adsorbed serum proteins for PC12 cell adhesion and growth on chitosan and collagen/chitosan surfaces
Source: Regen Biomater. 2018 Jul 17;5(5):261–73. doi: 10.1093/rb/rby017 (PMC6184651; doi:10.1093/rb/rby017)
Supplement: Supplementary Information [file rby017_supporting_information.docx]

**Supporting Information**

**Title:** A proteomics study to explore the role of adsorbed serum proteins for PC12 cell adhesion and growth on chitosan and collagen/chitosan surfaces

**Author:** Xiaoying Lü, Heng Zhang, Yan Huang, Yiwen Zhang

**Supplementary Table 1.** Proteins adsorbed on both chitosan and collagen/chitosan surfaces.

**Supplementary Table 2.** 26 proteins adsorbed only on chitosan surface.

**Supplementary Table 3.** 20 proteins adsorbed only on Collagen/chitosan surface.

**Supplementary Table 4.** The biological pathways that adsorbed proteins on the chitosan and collagen/chitosan surfaces involved in.

**Supplementary Table 5.** Pathways that adsorbed proteins involved as ligand- receptors binding.

**Supplementary Table 1.** Proteins adsorbed on both chitosan and collagen/chitosan surfaces.

| No. | Gene ID | Protein name | Chitosan film (%) | Collagen/chitosan film (%) |
| --- | --- | --- | --- | --- |
| 1 | 280717 | serum albumin | 25.161 | 32.100 |
| 2 | 280988 | alpha-2-HS-glycoprotein | 12.872 | 11.070 |
| 3 | 511735 | hemoglobin fetal subunit beta | 8.313 | 1.204 |
| 4 | 618039 | apolipoprotein C-II | 6.615 | 2.744 |
| 5 | 280699 | alpha-1-antiproteinase | 5.450 | 7.463 |
| 6 | 280948 | transthyretin | 4.959 | 5.267 |
| 7 | 512439 | hemoglobin subunit alpha | 4.952 | 6.302 |
| 8 | 497200 | alpha-1-acid glycoprotein | 3.919 | 4.792 |
| 9 |  | apolipoprotein A-I | 3.871 | 3.447 |
| 10 |  | serotransferrin | 2.822 | 3.106 |
| 11 | 515783 | tetranectin | 2.815 | 3.214 |
| 12 | 513856 | alpha-2-macroglobulin | 2.382 | 1.646 |
| 13 | 408009 | apolipoprotein C-III | 2.238 | 0.756 |
| 14 | 505394 | apolipoprotein A-II | 1.863 | 2.577 |
| 15 | 286804 | serpin A3-1 | 0.797 | 0.716 |
| 16 | 281631 | apolipoprotein A-I preproprotein | 0.581 | 0.298 |
| 17 |  | adiponectin | 0.568 | 0.564 |
| 18 |  | hemoglobin alpha chain | 0.499 | 0.761 |
| 19 | 280705 | serotransferrin | 0.432 | 2.946 |
| 20 | 506828 | Predicted: alpha-2-macroglobulin | 0.325 | 0.109 |
| 21 | 530076 | vitamin D-binding protein | 0.319 | 0.423 |
| 22 |  | prepro complement component | 0.301 | 0.341 |
| 23 | 504615 | fetuin-B | 0.271 | 0.525 |
| 24 |  | anti-testosterone antibody | 0.204 | 1.257 |
| 25 | 281370 | polyubiquitin-B | 0.193 | 0.638 |
| 26 |  | Ig heavy chain | 0.184 | 0.303 |
| 27 | 518955 | alpha-1B-glycoprotein | 0.173 | 0.135 |
| 28 | 513700 | inter-alpha-trypsin inhibitor heavy chain H4 | 0.162 | 0.158 |
| 29 | 511239 | protein HP-25 homolog 2 | 0.153 | 0.197 |
| 30 | 507525 | vitronectin | 0.135 | 0.012 |
| 31 | 537301 | apolipoprotein A-IV | 0.130 | 0.204 |
| 32 | 506890 | IGK protein | 0.129 | 0.125 |
| 33 | 281006 | beta-2-glycoprotein 1 | 0.121 | 0.216 |
| 34 | 540261 | antithrombin-III | 0.118 | 0.076 |
| 35 | 282164 | cathelicidin-1 | 0.102 | 0.077 |
| 36 | 616715 | protein HP-20 homolog | 0.097 | 0.130 |
| 37 | 539901 | E3 ubiquitin-protein ligase BRE1B | 0.095 | 0.170 |
| 38 | 280692 | haptoglobin | 0.089 | 0.097 |
| 39 |  | beta-lactoglobulin | 0.087 | 0.045 |
| 40 | 511240 | protein HP-25 homolog 1 | 0.080 | 0.109 |
| 41 | 280897 | plasminogen | 0.070 | 0.061 |
| 42 | 527553 | C-reactive protein | 0.065 | 0.030 |
| 43 | 534509 | hemopexin | 0.059 | 0.161 |
| 44 |  | bIGFBP-1 | 0.057 | 0.247 |
| 45 | 514076 | complement factor B | 0.053 | 0.068 |
| 46 | 617696 | similar to complement component 4A | 0.050 | 0.010 |
| 47 | 515150 | C4b-binding protein alpha chain | 0.048 | 0.102 |
| 48 | 514663 | leucine-rich alpha-2-glycoprotein | 0.038 | 0.021 |
| 49 | 281035 | factor XIIa inhibitor | 0.037 | 0.006 |
| 50 | 282522 | alpha-2-antiplasmin | 0.037 | 0.048 |
| 51 |  | lactotransferrin | 0.028 | 0.025 |
| 52 |  | prothrombin | 0.028 | 0.007 |
| 53 | 497203 | serpin A3-7 | 0.028 | 0.078 |
| 54 | 280740 | carbonic anhydrase 2 | 0.024 | 0.018 |
| 55 | 513197 | complement factor I | 0.023 | 0.017 |
| 56 |  | retinol-binding protein | 0.022 | 0.047 |
| 57 | 280816 | complement factor H | 0.019 | 0.029 |
| 58 | 281386 | pigment epithelium-derived factor | 0.018 | 0.005 |
| 59 | 280750 | clusterin preproprotein | 0.017 | 0.016 |
| 60 | 535077 | gelsolin isoform b | 0.016 | 0.017 |
| 61 | 280794 | fibronectin | 0.015 | 0.021 |
| 62 | 280685 | coagulation factor II (thrombin) | 0.014 | 0.037 |
| 63 | 505308 | serum amyloid A-4 protein | 0.013 | 0.042 |
| 64 | 494004 | PREDICTED: apolipoprotein B-100 | 0.012 | 0.001 |
| 65 |  | kininogen I | 0.011 | 0.021 |
| 66 |  | endopin 2C | 0.010 | 0.046 |
| 67 |  | immunoglobulin light chain variable region | 0.010 | 0.065 |
| 68 |  | complement component C4 | 0.009 | 0.025 |
| 69 | 527114 | angiotensinogen | 0.007 | 0.005 |
| 70 | 280787 | coagulation factor X | 0.006 | 0.005 |
| 71 | 512045 | complement C5 | 0.005 | 0.002 |
| 72 | 514194 | ceruloplasmin (ferroxidase) | 0.004 | 0.004 |
| 73 | 514588 | fibulin-1 | 0.004 | 0.003 |
| 74 |  | Kininogen- I | 0.002 | 0.003 |
| 75 | 280996 | protein AMBP | 0.002 | 0.016 |
| 76 | 526766 | complement component C9 | 0.001 | 0.001 |
| 77 | 517172 | protein Z-dependent protease inhibitor | 0.001 | 0.002 |
| 78 |  | inter-alpha (globulin) inhibitor H3 | 0.001 | 0.009 |

**Supplementary Table 2.** 26 proteins adsorbed only on chitosan surface.

| No. | Gene ID | Protein name | Relative abundance (%) |
| --- | --- | --- | --- |
| 1 |  | MHC class II antigen | 0.928 |
| 2 | 505478 | IGL@ protein | 0.802 |
| 3 | 404103 | spleen trypsin inhibitor I | 0.228 |
| 4 |  | MHC class II DR-beta chain | 0.194 |
| 5 |  | MHC class II DQA2 | 0.093 |
| 6 |  | BoLA-DRB3 | 0.088 |
| 7 | 613999 | hepatitis A virus cellular receptor 1 | 0.080 |
| 8 | 616035 | PREDICTED: serum amyloid A protein isoform 2 | 0.055 |
| 9 | 784932 | PREDICTED: endopin 2B-like | 0.054 |
| 10 | 281239 | insulin-like growth factor I preproprotein | 0.051 |
| 11 |  | immunoglobulin lambda light chain variable region | 0.031 |
| 12 | 338050 | plasma serine protease inhibitor | 0.021 |
| 13 |  | immunoglobulin heavy chain constant region | 0.018 |
| 14 | 519195 | PREDICTED: coiled-coil domain containing 81-like | 0.013 |
| 15 | 519737 | PREDICTED: complement factor H-related protein 2 | 0.013 |
| 16 | 282518 | thyroxine-binding globulin | 0.013 |
| 17 |  | Hageman factor | 0.013 |
| 18 | 281210 | glutathione peroxidase 3 | 0.012 |
| 19 | 505830 | APOM protein | 0.012 |
| 20 | 533307 | PREDICTED: tubulin beta chain | 0.010 |
| 21 | 280813 | hemoglobin subunit beta | 0.009 |
| 22 | 280820 | histidine-rich glycoprotein | 0.007 |
| 23 | 100125764 | heparin cofactor 2 | 0.005 |
| 24 | 280687 | coagulation factor V | 0.002 |
| 25 | 5852893 | hypothetical protein MGL_4276 | 0.002 |
| 26 |  | pantetheinase | 0.002 |
| Total | | | 2.756 |

**Supplementary Table 3.** 20 proteins adsorbed only on collagen/chitosan surface.

| No. | Gene ID | Protein name | Relative abundance (%) |
| --- | --- | --- | --- |
| 1 | 100272170 | serpin A3-2 | 0.139 |
| 2 |  | immunoglobulin lambda light chain | 0.110 |
| 3 | 444858 | eIF4GI protein isoform 1 | 0.076 |
| 4 |  | IgM heavy chain | 0.061 |
| 5 | 507790 | platelet factor 4 | 0.054 |
| 6 | 506412 | serum amyloid A protein | 0.046 |
| 7 | 281297 | mannose-binding protein C | 0.036 |
| 8 |  | thrombospondin-1 | 0.034 |
| 9 | 522073 | reticulocalbin-3 | 0.033 |
| 10 | 280849 | lysozyme C-2 | 0.033 |
| 11 | 280821 | immunoglobulin J chain | 0.019 |
| 12 | 445463 | ovarian and testicular apolipoprotein N | 0.010 |
| 13 | 508800 | zinc-alpha-2-glycoprotein | 0.007 |
| 14 | 280847 | lumican | 0.006 |
| 15 |  | prothrombin | 0.004 |
| 16 | 506011 | alpha-fetoprotein | 0.003 |
| 17 | 541281 | thrombospondin-4 | 0.003 |
| 18 | 505819 | mannan-binding lectin serine protease 2 | 0.002 |
| 19 | 282188 | collagen alpha-2(I) chain | 0.001 |
| 20 | 517356 | complement component C8 gamma chain | 0.001 |
| Total | | | 0.678 |

**Supplementary Table 4.** The biological pathways that adsorbed proteins on the chitosan and collagen/chitosan surfaces involved in.

|  | No. | Pathway | Contained protein | |
| --- | --- | --- | --- | --- |
|  |  |  | Chitosan film | Collagen/chitosan film |
| Proteins adsorbed on chitosan and collagen/chitosan surface involved | 1 | Complement and coagulation cascades | 20 | 17 |
|  | 2 | Systemic lupus erythematosus | 7 | 5 |
|  | 3 | PPAR signaling pathway | 5 | 5 |
|  | 4 | Focal adhesion | 2 | 4 |
|  | 5 | Regulation of actin cytoskeleton | 2 | 2 |
|  | 6 | Pathways in cancer | 2 | 1 |
|  | 7 | ECM-receptor interaction | 2 | 4 |
|  | 8 | Prion diseases | 2 | 3 |
|  | 9 | Neuroactive ligand-receptor interaction | 2 | 2 |
|  | 10 | Ribosome | 1 | 1 |
|  | 11 | Nitrogen metabolism | 1 | 1 |
|  | 12 | Small cell lung cancer | 1 | 1 |
|  | 13 | Renin-angiotensin system | 1 | 1 |
|  | 14 | Fc gamma R-mediated phagocytosis | 1 | 1 |
|  | 15 | Type Ⅱ diabetes mellitus | 1 | 1 |
|  | 16 | Adipocytokine signaling pathway | 1 | 1 |
| Proteins adsorbed only on chitosan surface involved | 1 | Allograft rejection | 2 |  |
|  | 2 | Type I diabetes mellitus | 2 |  |
|  | 3 | Autoimmune thyroid disease | 2 |  |
|  | 4 | Intestinal immune network for IgA production | 2 |  |
|  | 5 | Viral myocarditis | 2 |  |
|  | 6 | Asthma | 2 |  |
|  | 7 | Graft-versus-host disease | 2 |  |
|  | 8 | Antigen processing and presentation | 2 |  |
|  | 9 | Cell adhesion molecules (CAMs) | 2 |  |
|  | 10 | Pantothenate and CoA biosynthesis | 1 |  |
|  | 11 | mTOR signaling pathway | 1 |  |
|  | 12 | Gap junction | 1 |  |
|  | 13 | Dilated cardiomyopathy | 1 |  |
|  | 14 | Prostate cancer | 1 |  |
|  | 15 | Progesterone-mediated oocyte maturation | 1 |  |
|  | 16 | Glutathione metabolism | 1 |  |
|  | 17 | Arachidonic acid metabolism | 1 |  |
|  | 18 | Hypertrophic cardiomyopathy(HCM) | 1 |  |
|  | 19 | Oocyte meiosis | 1 |  |
|  | 20 | Long-term depression | 1 |  |
|  | 21 | Aldosterone-regulated sodium reabsorption | 1 |  |
|  | 22 | Melanoma | 1 |  |
|  | 23 | p53 signaling pathway | 1 |  |
|  | 24 | Glioma | 1 |  |
| Proteins adsorbed only on collagen/chitosan surface involved | 1 | TGF-beta signaling pathway |  | 1 |
|  | 2 | Chemokine signaling pathway |  | 1 |
|  | 3 | Cytokine-cytokine receptor interaction |  | 1 |

**Supplementary Table 5.** Pathways that adsorbed proteins involved as ligand- receptors binding.

| No. | Pathway | Contained protein | |
| --- | --- | --- | --- |
|  |  | Chitosan film | Collagen/chitosan film |
| 1 | Complement and coagulation cascades | 20 | 17 |
| 2 | Focal adhesion | 2 | 4 |
| 3 | Regulation of actin cytoskeleton | 2 | 2 |
| 4 | ECM-receptor interaction | 2 | 4 |
| 5 | Neuroactive ligand-receptor interaction | 2 | 2 |
| 6 | Adipocytokine signaling pathway | 1 | 1 |
| 7 | TGF-beta signaling pathway |  | 1 |
| 8 | Chemokine signaling pathway |  | 1 |
| 9 | Cytokine-cytokine receptor interaction |  | 1 |
